# Targeting the Opening of Mitochondrial Permeability Transition Pores Potentiates Nanoparticle Drug Delivery and Mitigates Cancer Metastasis

**DOI:** 10.1002/advs.202002834

**Published:** 2020-12-31

**Authors:** Xi Lin, Lian Li, Shujie Li, Qiuyi Li, Dandan Xie, Minglu Zhou, Yuan Huang

**Affiliations:** ^1^ Key Laboratory of Drug Targeting and Drug Delivery System (Ministry of Education) West China School of Pharmacy Sichuan University No. 17, Block 3, South Renmin Road Chengdu 610041 P.R. China

**Keywords:** cancer therapy, mitochondria targeting, MPTP opening, self‐assembly nanoparticle, tumor metastasis suppression

## Abstract

Mitochondria are highly involved in the metastasis of cancer cells. However, low permeability of mitochondria impedes the entry of anti‐cancer drugs. Here, a self‐assembled nanoparticle platform is designed that not only targets the DNA‐intercalating agent doxorubicin to mitochondria but also enhances the specific penetration by opening the mitochondrial permeability transition pores (MPTPs). With drastic improvement in mitochondrial uptake, the drug delivery system results in substantial mitochondrial impairment leading to amplified induction of apoptosis, depletion of energy supply, and inhibition of numerous metastasis‐associated proteins. As a consequence, the drug delivery system significantly inhibits the orthotopic tumor growth, and suppressed the metastasis of cancer cells detached from primary tumors. Additionally, the nanoparticle exhibits a potent effect on eradicating the metastasis of disseminated tumor cell from blood to lung. The results show that strategies of targeting mitochondria and unlocking MPTP are feasible and beneficial to mitigate both tumorigenesis and metastasis.

## Graphical abstract

A self‐assembled nanoparticle platform that escorts the DNA‐intercalating agent doxorubicin to the mitochondrial surface and enhances specific penetration by opening the mitochondrial permeability transition pores (MPTPs) is described. The improvement in the mitochondrial uptake results in an amplified induction of apoptosis, depletion of energy supply, inhibition of orthotopic tumor growth, and the suppression of cancer metastasis.

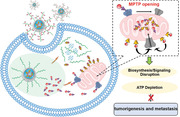

## 1 Introduction

Metastasis is a leading cause of over 90% of cancer death in clinic. Primary tumors continuously release disseminated tumor cells that migrate, seed, and colonize distant organs.^[^ ^1^ ^]^ Therefore, effective cancer treatment largely depends on concurrently destroying primary tumors and eradicating metastatic tumors at the secondary sites.^[^ ^2^ ^]^

Accumulating evidence has suggested that mitochondria may be a pharmacological target to mitigate both tumorigenesis and metastasis.^[^ ^3^ ^]^ Indeed, mitochondria actively participates in biosynthesis needed for tumor progression, and also has a well‐recognized role in empowering a cell to metastasize by generating adenosine triphosphate (ATP) as the fuel.^[^ ^4^ ^]^ In addition, a variety of pro‐survival proteins and signaling (e.g., Bcl‐1 and matrix metallopeptidase (MMP2)) that promote metastasis reside in mitochondria or are closely associated with mitochondrial functions.^[^ ^5^ ^]^ Thereby, specific mitochondrial damage can directly disrupt biosynthesis rendering primary tumor cells apoptotic, inhibiting relevant signaling, and cutting off the energy supply that supports the migration and invasion of metastatic cancer cells.

However, effectively delivering drugs to the mitochondria is a challenging task.^[^ ^6^ ^]^ To date, most of the mitochondria‐argeted agents contain a lipophilic cation (e.g., triphenyl phosphonium, TPP^+^) covalently attached to various bioactive compounds.^[^ ^7^ ^]^ The driving force of their selective uptake is the electrostatic interaction with the negative mitochondrial‐membrane‐potential ΔΨ (≈−150 to −180 mV). Yet, those TPP‐functionalized compounds lack efficacy in vivo. Typically, the mitochondria membrane is highly impermeable, leaving a majority of cationic targeting moieties remaining mainly on the membrane surface due to a limited effect of electrostatic interaction.^[^ ^7^, ^8^ ^]^ Moreover, the hydrophobicity and positive charge of these mitochondria targeted compounds consequently result in insufficient blood circulation and tumor accumulation, constituting another problem for systemic delivery.^[^ ^8^ ^]^ Thus, these findings call for innovative strategies to circumvent the obstacles to improving TPP‐drug therapeutics.

Targeting the opening of mitochondrial permeability transition pores (MPTPs) may be a new direction to enhance mitochondrial penetration. Since the transport of even small particles via the mitochondrial membrane is strictly governed by MPTPs, opening pores can allow an increase in the permeability of the mitochondrial membranes for molecules in close proximity to the mitochondrial area.^[^ ^9^ ^]^ Glycyrrhetinic acid (GA), an extract of licorice, has been reported to interact with the mitochondrial respiratory chain, resulting in the generation of hydrogen peroxide that triggers the opening of MPTPs.^[^ ^10^ ^]^ Although there has been no report of exploiting the MPTP opening to enhance mitochondrial delivery of anti‐cancer drugs so far, we assume GA might serve a dual role: 1) to escort drugs covalently linked to the GA to the mitochondria by targeting MPTP; and 2) to enhance the mitochondrial entry of TPP‐drug conjugates, attaching on mitochondria surface, by triggering MPTP opening.

**Figure 1:**
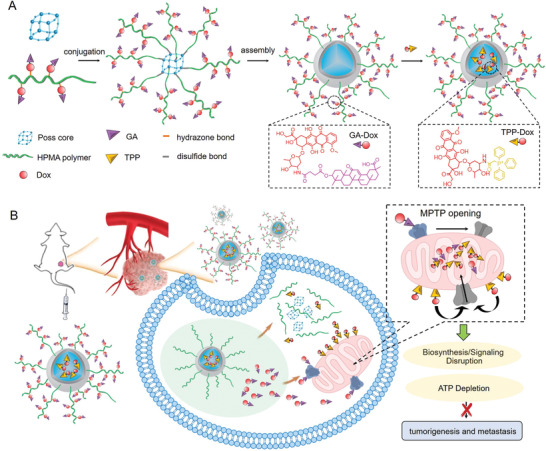
A) Synthetic procedure and B) schematic mechanism illustration of GD‐NP(TD) nanoparticle (NP) platform with GA‐Dox (GD) conjugated in the polymeric shell and TPP‐Dox (TD) encapsulated in the core.

Self‐assembled nanomedicine has drawn great attention as a feasible vehicle to deliver drugs to tumor sites and even subcellular organelles,^[^ ^11^ ^]^ and has also been used to treat cancer metastasis.^[^ ^12^ ^]^ Polyhedral oligomeric silsesquioxane (POSS) has been widely used as building blocks to form amphiphilic hybrid inorganic–organic materials that are not only capable of efficient hydrophobic drug encapsulation, but can also self‐assemble into nanoparticles in aqueous solutions.^[^ ^13^ ^]^ Thus, to deliver TPP‐drug in vivo and incorporate GA functions, a hybrid polymeric nanoparticle system self‐assembled from amphiphilic conjugates of an octaammonium POSS (POSS‐NH2) core and multiple *N*‐(2‐hydroxypropyl) methacrylamide (HPMA) polymers is developed. As shown in **Figure** **1**A, TPP‐functionalized doxorubicin (TPP‐Dox) is loaded in the POSS cage, which allows the shielding of the positive charge. The hydrophilic shell of HPMA polymers not only ensures adequate blood circulation and tumor accumulation of the delivery system, but also accommodates GA modified Dox (GA‐Dox) that targets MPTPs.

As illustrated in Figure 1B, after internalization into the lysosome of cancer cells, GA‐Dox is detached from the HPMA copolymer due to the breakage of a pH‐sensitive hydrazone linker in the acidic environment, and subsequently diffuses outside of the lysosome. Upon entry into the cytoplasm, a high concentration of glutathione (GSH) cleaves the disulfide bonds between POSS and HPMA polymer, leading to the disassembly of nanoparticles and release of the loaded TPP‐DOX from the cores. Then the TPP‐Dox can realize mitochondrial surface‐targeting by interaction with the mitochondrial membrane via electrostatic interaction. Our results reveal that the GA not only facilitated the GA‐Dox to accumulate in the mitochondria, but also performed as a key to unlock MPTP leading to the enhanced mitochondrial uptake of TPP‐Dox. Meanwhile, the GA did not exert an enhancing effect on the mitochondrial uptake of naive Dox, meaning that drug uptake through MPTP opening is specific and requires TPP‐mediated pre‐targeting on the mitochondria surface. With GA and TPP working collectively, the source (apoptosis initiation in primary tumor), motivation (pro‐metastasis signal inhibition), and energy (ATP depletion) of cancer cell metastasis are potently blocked.

## 2 Results and Discussion

### 2.1 Synthesis and Characterization

**Figure 2:**
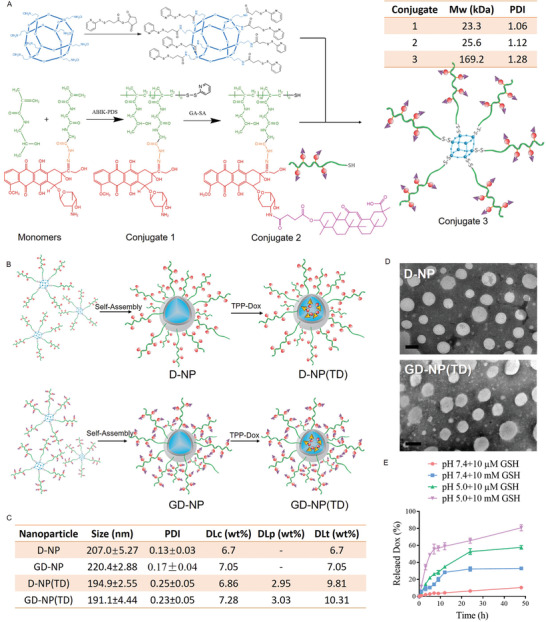
A) Synthesis and characterizations of amphiphilic conjugates of a POSS core and Dox‐loading HPMA polymers. B) Structure illustrations of D‐NP, GD‐NP, D‐NP(TD), and GD‐NP(TD). C) Characterizations of various NPs including size, PDI, covalently conjugated Dox loading (DLc), physically encapsulated Dox loading (DLp) and total Dox loading (DLt). D) Transmission electron micrographs of sphere morphologies of D‐NP and GD‐NP(TD). The scale bar is 200 nm. E) Drug release from GD‐NP(TD) over time after incubation with phosphate buffer (pH 7.4 or pH 5.0) containing different concentrations of GSH (10 µm and 10 mm).

As shown in **Figure** **2**A, the semitelechelic linear polymer‐Dox conjugate (Conjugate 1) was synthesized by radical solution polymerization of HPMA monomers and *N*‐methacryloylglycylglycyl‐hydrazide‐Dox (Ma‐GG‐NHN = Dox) monomers. Then GA was covalently attached to the Dox to obtain Conjugate 2. Afterward, the star‐like Conjugate 3 composed of a POSS core and multiple GA‐Dox‐loaded polymers was prepared by the reaction between pyridyldisulfanyl (PDS)‐functionalized POSS and thiol‐terminated Conjugate 2. The molecular weight (Mw) of Conjugate 2 and Conjugate 3 were 25.6 and 169.2 kDa, respectively, showing approximately 6–7 semitelechelic copolymer chains grafted to the POSS core. Due to the amphiphilic property, Conjugate 3 could self‐assemble into nanoparticles in the aqueous solution to fabricate GA‐Dox‐loaded nanoparticles (GD‐NP, structures illustrated in Figure 2B). Inside GD‐NP, the hydrophobic POSS created a particularly spatial environment that easily encapsulated the mitochondrial surface‐targeting compound of TPP‐Dox, generating a GD‐NP(TD) nanoparticle platform with GA‐Dox (GD) conjugated in the polymeric shell and TPP‐Dox (TD) loaded in the core. For various purposes, a series of control nanoparticles were also prepared, including non‐GA modified nanoparticle D‐NP(TD) and its counterpart D‐NP without encapsulation of TPP‐Dox, G‐NP(TD) nanoparticle that had GA attached to the polymeric shell, and TPP‐Dox encapsulated in the core and its non‐GA modified counterpart NP(TD).

GD‐NP(TD) showed a successful Dox load of ≈10 wt% and a sphere morphology with a narrow‐distributed size of around 200 nm (Figure 2C,D). Similar properties were also found in D‐NP, GD‐NP, and D‐NP(TD). To ensure the precise release of therapeutic payloads, intracellular stimuli were exploited. Typically, the lysosomal pH value in cancer cells decreases to pH 5.0–5.5 in comparison with that in blood circulation and normal tissues (pH 7.4). Additionally, tumor cytosol (in the concentration range of ≈2–10 mm GSH) has a far more reducing environment than the extracellular components (≈2–20 µm GSH). With acid‐responsive hydrazone bonds (linkage between GA‐Dox and HPMA polymer) and redox‐sensitive disulfide bonds (linkage between POSS core and Conjugate 2) embedded, GD‐NP(TD) exhibited a dual stimuli‐responsive drug release pattern (Figure 2E). Rapid drug release was found in the conditions of pH 5.0 (mimicking acidic lysosome) and 10 mm GSH (mimicking cell cytoplasm), whereas GD‐NP(TD) remained stable under the conditions of pH 7.4 (mimicking physiological environment) and 10 µm GSH (mimicking extracellular condition). The particle size of GD‐NP(TD) remained unchanged during a 24 h incubation in a PBS and cell culture medium containing 10% fetal bovine serum (Figure S1, Supporting Information), suggesting a good stability in physiological conditions.

### 2.2 GA Targets Mitochondria and Unlocks MPTP

**Figure 3:**
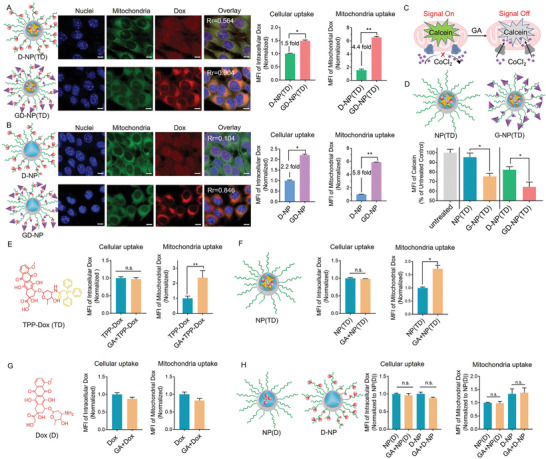
A) Comparison of D‐NP(TD) with GD‐NP(TD), and B) comparison of D‐NP with GD‐NP in their cell uptake and mitochondria uptake. A value of Rr, Pearson's correlation coefficient, close to 1 indicates reliable colocalization and vice versa. Confocal imaging of cellular uptake and mitochondria colocalization (left panels); Flow cytometry quantification results of cellular uptake (middle panels) and mitochondria uptake (right panels). C) Schematic mechanisms of investigating MPTP opening. D) Evaluation of the activities of MPTP opening by various nanoparticles. E–H) Cell uptake and mitochondria uptake of (E) TPP‐Dox, (F) NP(TD), (G) Dox, and (H) NP(D) and D‐NP in the absence and presence of GA. *n* = 3; *p < 0.05; **p < 0.01.

After intracellular release from GD‐NP(TD), Dox was expected to target mitochondria due to GA's capability to localize in mitochondria^[^ ^14^ ^]^ and TPP‐mediated electrostatic interaction with mitochondrial membrane.^[^ ^7^ ^]^ Meanwhile, a further enhancement of Dox uptake by mitochondria was supposed to occur because GA unlocks the MPTP thereby facilitating the mitochondrial entry of TPP‐Dox that pre‐targeted mitochondria. To demonstrate this hypothesis, we first investigated the overall effect of GA on increasing the mitochondrial uptake of GD‐NP(TD) (**Figure** **3**A). As compared with D‐NP(TD), GD‐NP(TD) exhibited a 1.5‐fold increase in cell internalization, and a higher increase of 4.4‐fold in mitochondrial uptake. This was further confirmed by confocal microscopy imaging: only a small proportion of Dox delivered by D‐NP(TD) arrived into the mitochondria (Rr = 0.546 between mitochondria and Dox) while GD‐NP(TD) delivered a substantial amount of Dox into the mitochondria (Rr = 0.904). The augmented mitochondrial uptake of Dox from GD‐NP(TD) was partially ascribed to a direct targeting effect of GA that escorted GA‐Dox to mitochondria, because GD‐NP resulted in a profound co‐localization degree of Dox with mitochondria (Rr = 0.846) while a bare mitochondrial uptake of Dox from D‐NP (Rr = 0.104) was observed (Figure 3B). Interestingly, surface modification with GA could increase the cell uptake of GD‐NP(TD) and GD‐NP as compared with their non‐GA modified counterparts (D‐NP(TD), D‐NP), probably due to the interaction between GA and its receptor protein kinase C (PKC) *α* that is highly expressed in cancer cells.^[^ ^14^ ^]^ It should be noted that this increased cellular uptake did not play a decisive effect to the GA‐mediated increment of mitochondrial uptake, because GA modification only mildly enhanced the cell uptake by approximately twofold but more efficiently increased the mitochondria uptake by nearly fivefold.

Another factor that contributed to the GA‐enhanced mitochondrial uptake might be the modulation of the MPTP opening that accelerated the mitochondrial entry of TPP‐Dox by attaching onto the mitochondria surface. To evaluate the effect of GA on the MPTP opening, we pre‐loaded the cells with fluorescent probe Calcein which distributed into the cytoplasm and mitochondria within the cells. Then the cells were treated with CoCl_2_ that could quench the fluorescence of Calcein. Typically, the MPTPs are closed to exclude CoCl_2_ so that the Calcein still exhibited fluorescence in the mitochondria but not in the cytoplasm. Once the opening of MPTP is triggered, it will allow CoCl_2_ entry into the mitochondria, turning off the Calecin signal. Thus, a reduction in Calcein fluorescence indicates the opening of MPTP (Figure 3C). As shown in Figure 3D, GA‐functionalized nanoparticles (G‐NP(TD) and GD‐NP(TD)) resulted in a significant decrease in Calcein fluorescence as compared with the corresponding counterparts (NP(TD) and D‐NP(TD)), demonstrating that the GA did induce the opening of MPTPs. Thus, a dual role of mitochondrial targeting and MPTP opening by GA was confirmed.

Having demonstrated that the GA targeted mitochondria and unlocked the MPTP, we next sought to investigate whether the GA could assist in the entry of TPP‐Dox into the mitochondria. As shown in Figure 3E, GA did not increase the cell uptake of TPP‐Dox, but dramatically increased the mitochondrial permeability to TPP‐Dox, suggesting the presence of GA favored mitochondrial targeting by TPP‐Dox. To further evaluate the role of GA within a nanoparticle system, we also fabricated NP(TD) that encapsulated TPP‐Dox. In a similar trend, the presence of free GA had no impact on cellular uptake of NP(TD), but significantly enhanced the mitochondrial accumulation of TPP‐Dox delivered by NP(TD) (Figure 3F). Interestingly, free GA was unable to increase the mitochondrial uptake of naive Dox (Figure 3G). In addition, free GA also failed to increase the mitochondrial uptake of Dox either when loaded inside the NP(D) or conjugated to polymeric shell of D‐NP (Figure 3H). These results demonstrated an indispensable role of TPP in the GA‐facilitated mitochondrial uptake of TPP‐Dox, and suggested that mitochondrial uptake through the MPTP opening is specific and necessarily requires TPP‐mediated pre‐targeting onto the mitochondria surface.

Collectively, we demonstrated that, in the Dox delivery system of GD‐NP(TD), GA exerts multifunctional effects: it not only directed GA‐Dox to target the mitochondria, but also triggered the opening of MPTP allowing for a further enhancement of the mitochondrial uptake of TPP‐Dox.

### 2.3 GD‐NP(TD) Enhances Apoptosis and Depletes ATP

**Figure 4:**
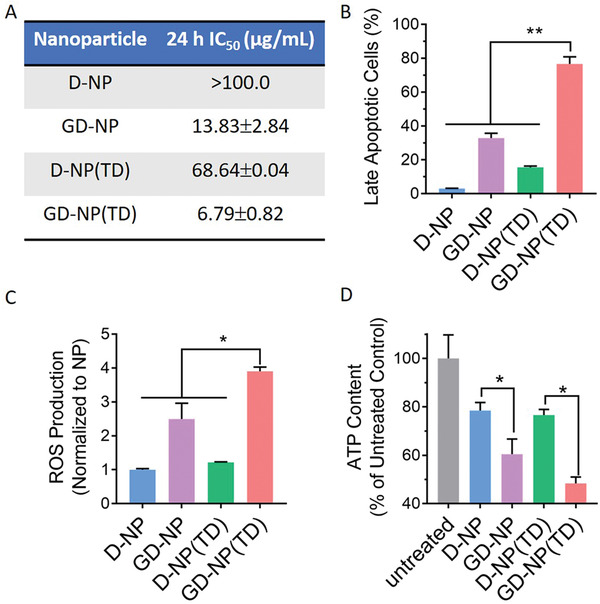
A) Cytotoxicity, B) late apoptosis induction, C) ROS production, and D) ATP depletion in tumor cells treated with various nanoparticles. *n* = 3; *p < 0.05; **p<0.01.

Mitochondrial DNA plays a crucial role in normal mitochondrial function and cell proliferation.^[^ ^15^ ^]^ We next investigated whether the enhanced delivery of DNA‐intercalating Dox to the mitochondria could translate into enhanced cell death and mitochondria dysfunction. Indeed, as compared with D‐NP, GD‐NP and D‐NP(TD), and GD‐NP(TD) with the highest mitochondrial uptake exerted significantly higher cytotoxicity (**Figure** **4**A) and resulted in a considerable increase in the number of cells undergoing late apoptosis (Figure 4B). Figure S2, Supporting Information, showed the cytotoxicity of GD‐NP(TD) on normal human umbilical vein endothelial cells (HUVEC). Even at the highest Dox concentration of 6.25 mg mL^−1^ which was close to the IC50 value of GD‐NP(TD) in 4T1 cells, the viability of HUVEC was still above 80%, suggesting HUVECs were less sensitive to GD‐NP(TD) than 4T1 cancer cells. In addition, although GA had inhibitory effects on tumor proliferation at a high concentration,^[^ ^14^ ^]^ GA‐modified blank nanoparticle (G‐NP) did not result in any inhibition of 4T1 cell growth while GA‐modified nanoparticle loaded with Dox (G‐NP(TD), GD‐NP, and GD‐NP(TD)) showed variable degrees of cytotoxicities (Figure S3, Supporting Information), suggesting that the cell death in this study was caused by the GA‐mediated mitochondrial targeting of Dox rather than the GA itself.

After arriving inside the mitochondria, Dox not only impairs the synthesis of mitochondrial DNA, but also sequentially interreacts with cytochrome P450 reductase to generate superoxide and hydrogen peroxide, interferes with the electron transfer in the electron transfer chain, and enhances the reactive oxygen species (ROS) accumulation. As mitochondria are a major source of ROS, elevation of the intracellular ROS is one of the characteristics of mitochondrial damage and is closely related to cell death.^[^ ^16^ ^]^ As shown in Figure 4C, GD‐NP(TD) also resulted in a significantly higher generation of intracellular ROS than D‐NP, GD‐NP, and D‐NP(TD), which correlated well with its ability to localize mitochondria and induce cytotoxicity.

As the powerhouse of cells, mitochondria generate ATP by aerobic glycolysis to provide energy for cell activities including proliferation and migration.^[^ ^17^ ^]^ Thus, the dysfunction of mitochondria highly affects the ATP production. Similarly, the best outcome of ATP depletion was achieved by GD‐NP(TD) among all tested groups as shown in Figure 4D, very likely because GD‐NP(TD) delivered the largest amount of Dox into the mitochondria and caused substantial mitochondrial damage.

### 2.4 GD‐NP(TD) Exerts Potent Anti‐Metastasis Activity

Cancer metastasis includes the growth and spreading of a bunch of active and energetic tumor cells to distal sites. Although Dox, a DNA damaging agent, mainly takes effect in the cell nucleus, it can also intercalate within the mitochondrial DNA resulting in mitochondrial dysfunction.^[^ ^18^ ^]^ In this study, we intended to deliver Dox to the mitochondria because the mitochondria are closely associated with cancer metastasis: 1) mitochondria actively participate in the biosynthesis needed for tumor progression; 2) numerous metastasis associated proteins reside in the mitochondria; 3) mitochondria provide energy that support the migration and invasion of metastatic cancer cells.^[^ ^19^ ^]^ Thus, targeting Dox to the mitochondria exhibits a unique advantage against cancer metastasis.

**Figure 5:**
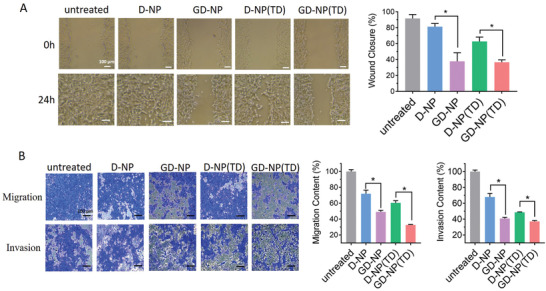
A) Wound healing, and B) migration and invasion after tumor cells were treated with various nanoparticles. *n* = 3; *p < 0.05; **p < 0.01.

With the ability to elicit apoptosis and deplete ATP by targeting and impairing mitochondria as demonstrated above, we next investigated whether GD‐NP(TD) could inhibit metastasis of cancer cells in vitro. To observe the effects on lateral tumor metastasis, we conducted a scratch‐wound assay (**Figure** **5**A). After 24 h treatment, the scratch wound was completed healed by untreated cells. While the anti‐metastatic effects from D‐NP and D‐NP(TD) were moderate and limited, GD‐NP and GD‐NP(TD) both resulted in a significant inhibition on the wound closure. In another experiment that measured the longitudinal cell migration and invasion, a similar inhibition trend of cell invasion was observed (Figure 5B). Furthermore, GD‐NP(TD) showed the highest suppression degree of cell migration among all tested treatments (Figure 5B). Collectively, GD‐NP(TD) exerted potent anti‐metastatic activities both laterally and longitudinally.

### 2.5 GD‐NP(TD) Suppresses Primary Tumor and Inhibits Tumor Metastasis

**Figure 6:**
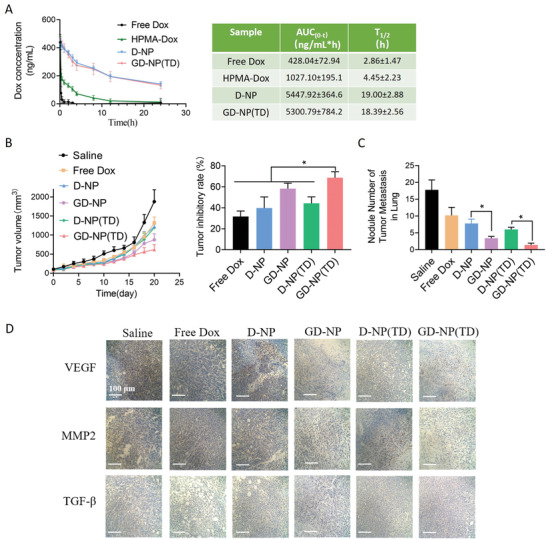
A) Plasma concentration profiles and pharmacokinetic parameters of free Dox, HPMA copolymer‐Dox conjugate, D‐NP, and GD‐NP(TD) after an intravenous injection in mice. B) Growth of tumor volume and endpoint inhibitory rate after the tumor‐bearing mice were treated with various nanoparticles. C) The nodule number of tumor metastasis in lungs at endpoints of the treatments. D) Representative images of immunohistochemical staining of MMP2, VEGF, and TGF‐*β* in primary tumors treated with various nanoparticles. *n* = 5; *p < 0.05; **p < 0.01.

An initial prerequisite for subcellular targeting, MPTPs opening, and mitochondrial damage by GD‐NP(TD) in vivo is the sufficient circulation half‐life that ensures contacts with cancer cells in the primary tumor and circulating tumor cells in the blood. Pharmacokinetic results in **Figure** **6**A showed that as compared with free Dox (*T*_1/2_ = 2.86 h) that was eliminated rapidly from the body, Dox that was conjugated to the HPMA copolymer (HPMA‐Dox, Mw = 22.3 kDa) had an extended circulation time (*T*_1/2_ = 4.45 h). As expected, with a size over the renal threshold (45 kDa), D‐NP self‐assembled from multiple amphiphilic conjugates of a POSS core and Dox‐loading HPMA polymers drastically prolonged its circulation (*T*_1/2_ = 19.00 h). In addition, GD‐NP(TD) had a similar pharmacokinetic profile (*T*_1/2_ = 18.39 h) with D‐NP, suggesting further GA modification and TPP‐Dox encapsulation did not affect the relatively long‐circulating behavior of GD‐NP(TD) in vivo.

To evaluate the in vivo therapeutic efficacy of GD‐NP(TD), 4T1 orthotopic tumor bearing mice models were intravenously treated when the primary tumor volume increased to 100 mm^3^. Results in Figure 6B showed that GD‐NP(TD) considerably suppressed the tumor growth with the inhibition rate of 68.7% at the endpoint, which was superior to other treatments including free Dox (31.6%), D‐NP (39.7%), D‐NP(TD) (44.3%), and GD‐NP (58.3%). Although D‐NP had a greatly prolonged circulation than free Dox, their effects on tumor inhibition were comparable. As compared with D‐NP, GA modification significantly improved the therapeutic outcome of GD‐NP, highlighting the necessity for further delivering Dox to subcellular mitochondria. Moreover, GA modification also resulted in the strongest tumor inhibition potency of GD‐NP(TD) that further encapsulated TPP‐Dox, while treatment with D‐NP(TD) was less satisfactory. This could be explained by the complementary mechanism demonstrated above: GA not only targeted GA‐Dox to mitochondria, but also enhanced the mitochondrial uptake of TPP‐Dox via unlocking the MPTPs.

Of note, GD‐NP(TD) also significantly reduced the cancer metastasis from primary tumors to lungs as compared with other controls (Figure 6C). The substantially enhanced apoptosis and energy depletion caused by GD‐NP(TD) mediated mitochondrial impairment could partially account for this phenomenon. In addition, we have also found that GD‐NP(TD) considerably downregulated the expressions of vascular endothelial growth factor (VEGF), MMP2, and transforming growth factor beta (TGF‐*β*) in primary tumors (Figure 6D). These proteins were reported to be closely engaged in promoting cancer metastasis, and could be negatively regulated in response to excessive ROS.^[^ ^20^ ^]^ Thus, GD‐NP(TD) could also inhibit numerous metastasis‐associated proteins through elevating intracellular ROS production as a result of mitochondrial dysfunction. These results emphasized the great feasibility of targeting mitochondria to mitigate both tumorigenesis and metastasis, which benefited from multiple downstream pathways including apoptosis induction, energy depletion, and pro‐metastasis signal inhibition. In addition, GD‐NP(TD) caused no body weight loss, cardiotoxicity, or liver and kidney injuries during the treatment, and exerted potent lung protection against cancer cell metastasis (Figure S4, Supporting Information), demonstrating the safety and efficiency of this treatment.

### 2.6 GD‐NP(TD) Inhibits Disseminated Tumor

**Figure 7:**
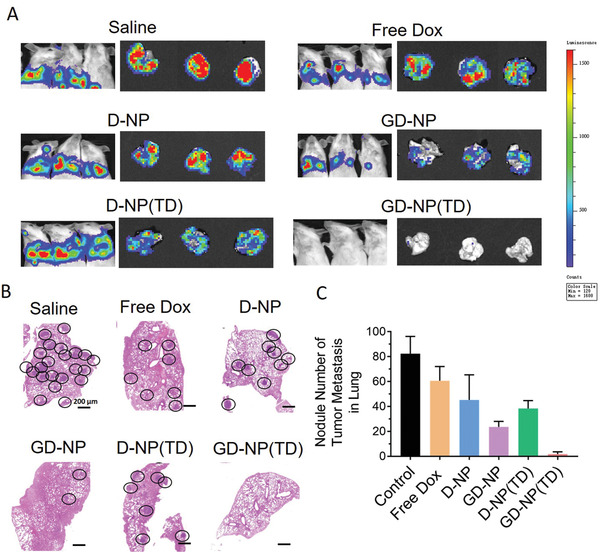
A) Bioluminescence images of 4T1‐Luc cells in mice and extracted lungs after 4T1‐Luc metastasis tumor‐bearing mice were treated with various nanoparticles. B) Hematoxylin–eosin histology analysis of lung lobe sections from mice at the endpoint of various treatments. C) The average number of metastatic tumor nodules in lungs from mice at the endpoint of various treatments. *n* = 5; *p < 0.05; **p < 0.01.

Finally, we investigated whether GD‐NP(TD) was capable of inhibiting the disseminated tumors that metastasized to lungs. 4T1‐Luc cells that exhibited bioluminescence were intravenously injected into BALB/c mice on day 0. Different treatments started on day 3, and were given every 3 days for four cycles. On day 21, in vivo bioluminescence in mice and ex vivo bioluminescence in extracted lung tissues were imaged (**Figure** **7**A). As compared with saline‐treated mice that had obvious establishment of lung metastasis, free Dox and D‐NP treated mice showed similarly strong bioluminescence and failed to mediate metastasis suppression. Additionally, D‐NP(TD) only slightly attenuated metastasis formation in lung. Although decreased bioluminescence in lung was observed in GD‐NP treated mice, the effect of GD‐NP was still limited to completely eradicate tumor metastasis. Markedly, no visible bioluminescence was found in mice treated with GD‐NP(TD), indicating the significant anti‐metastasis efficiency. This was further confirmed by the hematoxylin–eosin histology analysis (Figure 7B) and number count of metastatic tumor nodules (Figure 7C) in lung lobe sections from mice at the endpoint of various treatments. In consistency with bioluminescence images, GD‐NP(TD) resulted in bare formation of metastatic nodules or lesions in lung while the rest of groups had various degrees of lung metastasis. Accordingly, the metastasis suppression of disseminated tumor cells highly correlated with the capacity of nanoparticles to deliver Dox to the mitochondria. Of note, GD‐NP(TD) simultaneously escorted GA‐Dox to target mitochondria and triggered the opening of MPTPs to facilitate the mitochondrial entry of encapsulated TPP‐Dox, leading to significant mitochondrial impairment. Consequently, the source, motivation, and energy that support tumor metastasis were blocked by the apoptosis initiation in primary tumor, pro‐metastasis signal inhibition, and ATP depletion, respectively, thereby resulting in an almost complete removal of tumor metastasis.

## 3 Conclusion

We developed a self‐assembled nanoparticle platform with GA‐Dox conjugated on shell and TPP‐Dox encapsulated in the core. The fabricated therapy of GD‐NP(TD) simultaneously escorted GA‐Dox to target the mitochondria and triggered the opening of MPTPs to specifically facilitate the mitochondrial entry of encapsulated TPP‐Dox. Both GA and TPP modifications played indispensable roles in the drastic improvement of mitochondria uptake. Consequently, GD‐NP(TD) resulted in significant mitochondrial impairment leading to enhanced apoptosis induction, ROS generation, ATP depletion, and the inhibition of numerous metastasis‐associated proteins. As a result, GD‐NP(TD) not only significantly inhibited the growth of the primary tumor, but also suppressed the metastasis of tumor cells in the lung. Furthermore, GD‐NP(TD) was even able to repress the in vivo metastasis formation of disseminated tumor cell in lungs. In summary, here we provide proof of concept that strategies of targeting mitochondria and unlocking MPTP could potentiate nanoparticle drug delivery and mitigate cancer metastasis.

## 4 Experimental Section

##### Materials

Doxorubicin hydrochloride (Dox·HCl) was purchased from Dalian Meilun Biotech Co., Ltd. (Dalian, China). GA was purchased from J&K Co. Ltd. (Shanghai, China). Initiator ABIK‐PDS was synthesized according to a previous report. POSS‐NH2 was purchased from Hybrid Plastics (Hattiesburg, USA). Matrigel was purchased from BD Biosciences (Bedford, USA). 3‐(4,5‐dimethyl‐2‐tetrazolyl)‐2,5‐diphenyl‐2H tetrazolium bromide (MTT), 4’,6‐diamidino‐2‐phenylindole (DAPI) and reduced GSH, were purchased from Sigma‐Aldrich (St. Louis, MO, USA). MitoTracker Green was purchased from Invitrogen (Carlsbad, CA, USA). BCA Protein Assay Kit, SDS‐PAGE Gel Quick Preparation Kit, Mitochondrial Permeability Transition Pore Assay Kit, Cell Mitochondria Isolation Kit and Reactive Oxygen Species Assay Kit were purchased from Beyotime Institute of Biotechnology (Shanghai, China). FITC Annexin V Apoptosis Detection Kit with 7‐AAD were purchased from BioLegend. Comonomer HPMA and *N*‐methacryloylglycylglycyl‐hydrazide‐doxorubicin (MA‐GG‐NHN = Dox) were synthesized according to previous method.^[^ ^21^ ^]^ All other chemicals and reagents were of analytical grade. All other chemicals were bought from Energy Chemical (Shanghai, China).

##### Synthesis of Semitelechelic Conjugate 1

The thiol‐terminated HPMA polymer‐Dox conjugate (Conjugate 1, Figure 2A) was synthesized via two steps. Briefly, HPMA copolymer‐Dox conjugate containing 2‐PDS‐terminal was prepared by radical precipitation polymerization in dimethyl sulfoxide (initiator ABIK‐PDS, 5 wt%; monomer concentration 12.5 wt%; molar ratio HPMA/MA‐GG‐NHN = Dox was 70:30). The reaction mixture was saturated with nitrogen, and then stirred at 70 °C for 24 h in the dark. The copolymer was obtained from the polymerization solution by precipitation in acetone and then purified by dialysis against distilled water for one day to remove low Mw impurities. Then DL‐dithiothreitol was added into the copolymer and stirred softly at room temperature for 30 min. After being purified by dialysis and freeze‐dried, the thiol‐terminated Conjugate 1 was obtained as a pink powder.

##### Synthesis of Semitelechelic Conjugate 2

The thiol‐terminated HPMA polymer‐Dox‐GA conjugate (Conjugate 2, Figure 2A) was synthesized via two steps. First, GA (2.5 mmol), succinyl anhydride (10.0 mmol), and 4‐dimethylaminopyridine (5 mmol) were dissolved in dichloromethane and refluxed at 40 °C for 12 h. The mixture was washed with saturated sodium chloride solution and concentrated. Pure product was obtained after recrystallization from ethanol as a white powder to obtain GA succinic derivative (GA‐SA). Then GA‐SA (0.276 mmol) was activated by 1‐(3‐dimethylaminopropyl)‐3‐ethylcarbodiimide hydrochloride (EDC·HCl, 0.414 mmol) and N‐hydroxysuccinimide (NHS, 0.414 mmol), and then the mixture was stirred at room temperature for 3 h, followed by the reaction with the Conjugate 1 for 24 h in dark. The conjugate was further purified by dialysis against distilled water for 48 h. After being filtered through a 0.22 µm microporous membrane and freeze‐dried, Conjugate 2 was obtained as a pink powder.

##### Synthesis of Star‐Like Conjugate 3

The star‐like conjugates (Conjugate 3, Figure 2A) composed of a POSS core and multiple copies of attached Conjugate 2 were prepared by a reaction between PDS‐functionalized POSS (POSS‐PDS) and thiol‐terminated Conjugate 2. POSS‐PDS was synthesized as described previously.^[^ ^22^ ^]^ Conjugate 2 (10 µmol SH groups), dissolved in dimethyl sulfoxide (DMSO), was added dropwise to a stirred solution of POSS‐PDS (8.13 µmol PDS groups) in DMSO under argon atmosphere. After a 4 h reaction, the mixture was diluted with methanol and the products were purified by gel filtration (Sephadex LH‐20, methanol).

##### Preparation of Self‐Assembled Nanoparticle GD‐NP

Conjugate 3 (30 mg) was dissolved in 2 mL of DMSO and stirred for 2 h. The solution was then added dropwise into deionized water under sonication and stirred for 4 h, and then dialyzed against deionized water using a dialysis bag (MWCO 14 000) for 24 h. Self‐assembled GD‐NP was obtained after freeze‐drying. D‐NP that self‐assembled from star‐like conjugates of a POSS core and multiple Conjugate 1s was prepared following the same procedure.

##### Preparation of Self‐Assembled TPP‐Dox Encapsulated Nanoparticle GD‐NP(TD)

For the synthesis of TPP‐Dox, Dox·HCl (0.09 mmol) and triethylamine (12.5 µL) were dissolved in dimethyl formamide (DMF) and stirred for 2 h. Then, 3‐carboxypropyltriphenylphosphonium bromide (0.09mmol), activated by NHS (0.149 mmol) and EDCI⋅HCL (0.05 mmol) in DMF, was added dropwise into the mixture and stirred for 24 h. The crude product was obtained by precipitation in diethyl ether and further purified by column chromatography (silica gel, 100–200 mesh, dichloromethane: methanol = 10:1). The pure product of TPP‐Dox was obtained as a red powder. For the preparation of GD‐NP(TD), Conjugate 3 (30 mg) and TPP‐Dox (6 mg) was dissolved in 2 mL of DMSO and stirred for 2 h. The mixture was added dropwise into deionized water under sonication and stirred for 4 h. GD‐NP(TD) was then obtained after purification by dialysis and was freeze‐dried as a pink powder. D‐NP(TD) was also prepared following the same procedure.

##### Preparation of Self‐Assembled TPP‐Dox Encapsulated Nanoparticle G‐NP(TD)

The thiol‐terminated HPMA polymer‐GA conjugate was synthesized via three steps. First, HPMA copolymer containing 2‐PDS‐terminal were prepared by radical precipitation polymerization in dimethyl sulfoxide (containing: initiator ABIK‐PDS, 5 wt%; monomer concentration 12.5 wt%; molar ratio HPMA/MA‐GG‐NHN2 was 70:30). The reaction mixture was saturated with nitrogen, and then stirred at 70 °C for 24 h in the dark. The copolymer was obtained from a polymerization solution by precipitating it in acetone and then purifying it by dialysis against distilled water for one day to remove low Mw impurities. Then, GA (2.5 mmol) and triethylamine were dissolved in dichloromethane, and then levulinic acid (LA, 2.5 mmol), activated by isopropyl chloroformate (2.5 mmol), was added into mixture, and stirred for 2 h. After being washed with saturated ammonium chloride and sodium chloride solution, the GA levulinic derivative (GA‐LA) was obtained and purified by column chromatography. Finally, the thiol‐terminated HPMA polymer‐GA conjugate was obtained by the reaction of HPMA copolymer (containing 0.08 mmol NH2 groups) with GA‐LA (0.24 mmol) in methanol, and then the acetic acid was added into the mixture and stirred at room temperature for 24 h. The product was obtained from mixture by precipitation in diethyl ether and then dialysis purification. The preparation process of G‐NP(TD) was the same as the GD‐NP(TD).

##### Characterization of Conjugates and Nanoparticles

The molecular weight and poly dispersity index (PDI) of conjugates were estimated by fast protein liquid chromatography (AKTA FPLC) system (Amersham Biosciences, NJ). The dynamic light scanning size (DLS) and zeta potential of nanoparticles were measured on a Zetasizer Nano ZS90 (Malvern Instruments, UK). The morphology was observed via transmission electron microscopy (TEM, H‐600, Hitachi, Japan). The content of the thiol groups and Dox in nanoparticles were quantified by UV–Vis analysis at 343 and 480 nm as previously reported.

##### Drug Release Evaluation

The release profiles of nanoparticles were investigated by a dialysis method. 1 mL of nanoparticle at a 3 mg mL^−1^ concentration was placed into the dialysis bag (MWCO 14 000) and incubated at 37 °C under various buffered conditions (pH 7.4/10 µm GSH, pH 7.4/10 mm GSH, pH 5.0/10 µm GSH, and pH 5.0/10 mm GSH). At predetermined time points, 500 µL of the solution was collected and an equal volume of fresh medium was added. The drug concentration was determined using a Varioskan Flash Multimode Reader.

##### Cell Culture

4T1 murine breast cancer cells (iCell, Shanghai, China) were cultured in RPMI‐1640 medium supplemented with 10% fetal bovine serum and 1% penicillin−streptomycin in 5% CO_2_ at 37 °C in a humidified incubator. All experiments were performed on cells in the logarithmic growth phase.

##### Cell Uptake Measurement

4T1 cells were seeded in 12‐well plates incubated for 24 h. After being treated with various nanoparticles (equivalent to a Dox dose of 10 µg mL^−1^) for 12 h, the medium was removed and washed with PBS. Then the cells were harvested and quantitative analysis of the cellular uptake was measured by flow cytometric analysis. The mean fluorescence intensity of 1 × 10^4^ cells was recorded for each sample.

##### Mitochondrial Uptake Measurement

4T1 cells were seeded in 6‐well plates incubated for 24 h. After being treated with various nanoparticles (equivalent to a Dox dose of 10 µg mL^−1^) for 12 h, the medium was removed and washed with PBS. The cells were harvested and the mitochondria were isolated according to the cell mitochondria isolation kit, followed by flow cytometric analysis which was done immediately. The mean fluorescence intensity of 1 × 10^4^ mitochondria was recorded for each sample. For the observation of mitochondria targeting by confocal laser scanning microscopy, 4T1 cells were seeded on the coverslips, and then treated as described above. After being washed with cold PBS, cells were stained with MitoTracker Green for 30 min at 37 °C, and then fixed with 4% paraformaldehyde and stained with DAPI (5 µg mL^−1^) for 5 min. The fluorescence images were captured by confocal laser scanning microscopy.

##### MPTP Opening Assay

The opening of MPTP was evaluated using the MPTP assay kit (Beyotime, China). 4T1 cells were seeded in 12‐well plates incubated for 24 h. Then cells were treated with various nanoparticles (equivalent to a Dox dose of 10 µg mL^−1^) for 12 h. According to the kit instruction, the cells were harvested and stained with Calcein AM and CoCl_2_ solution for 40 min. The fluorescence intensity of each sample was determined using flow cytometry.

##### Cytotoxicity Assay

The cytotoxicity of nanoparticles against 4T1 cells was evaluated by MTT assay. The cells were seeded in 96‐well plates for 24 h. Then cells were treated with various nanoparticles at a series of Dox concentrations for 24 h. Afterward, MTT solution (5 mg mL^−1^, 20 µL) was added to each well and further incubated for another 4 h. The medium in each well was then removed and replaced by 180 µL DMSO to dissolve the formazan crystals. Absorbance at 570 nm was recorded by a microplate reader. The cell viability was calculated according to the following equation: (T−B)/(C−B) × 100% (T is the absorption value of the treatment group; C is the absorption value of the untreated control group; B refers to the absorption value of the culture medium.)

##### Cell Apoptosis Assay

Cell apoptosis was determined using the FITC Annexin V Apoptosis Detection Kit with 7‐AAD. 4T1 cells were treated with various nanoparticles (equivalent Dox dose, 5 µg mL^−1^) for 24 h. According to the kit instruction, the cells were harvested and stained with FITC Annexin V Apoptosis Detection Kit with 7‐AAD. The fluorescence intensities of each sample were determined using flow cytometry.

##### Intracellular ROS Measurement

Intracellular ROS levels were determined using the Reactive Oxygen Species Assay Kit. 4T1 cells were treated with various nanoparticles (equivalent Dox dose, 10 µg mL^−1^) for 24 h. According to the kit instruction, the cells were harvested and stained with ROS reactive agent (DCFH‐DA, 10 µm) in the kit. The fluorescence intensities of each sample were determined using flow cytometry.

##### Intracellular ATP Measurement

The intracellular ATP levels were measured using a luciferase‐based ATP assay kit (Beyotime, China). 4T1 cells were treated with various nanoparticles (equivalent Dox dose, 10 µg mL^−1^) for 24 h. Then cells were lysed and centrifuged at 12 000 g for 5 min. The supernatant was collected and incubated with the ATP detection working dilution in a ratio of 1:5. The luminescence intensity was detected by microplate reader.

##### Scratch‐Wound Assay

The scratch‐wound assay was used to evaluate the lateral cell migration. 4T1 cells were seeded in 6‐well plates. When the cell coverage reached 80–90%, scratch wounds were generated with a 200 µL pipet tip. Then cells were treated with various nanoparticles (equivalent Dox dose, 2 µg mL^−1^) for 24 h. Afterward, the images of wound healing were captured by microscope (IX81, Olympus, Japan). The migration distance of each group was analyzed by Image‐J software to calculate the degrees of wound healing.

##### Migration and Invasion Assay

The longitudinal cell migration and invasion was determined using a trans‐well method. For cell migration assay, 100 µL of serum‐free media with 1 × 10^5^ 4T1 cells were seeded onto the top chamber of inserts (24‐well, pore size, 8 µm, Millipore). For cell invasion assay, 20 µL of Matrigel (1.5 mg mL^−1^ diluted with serum free DMEM) was added to top chamber of inserts and incubated at 37 °C for 4 h, and then 100 µL of serum‐free media with 1 × 10^5^ 4T1 cells were added to the inserts. Afterward, 600 µL of cultured media with 10% FBS was added to the 24‐plate well. Nanoparticles (equivalent Dox dose, 2 µg mL^−1^) were added to the upper chambers of inserts. The untreated cells were performed as negative control. After 24 h, the migrated or invaded cells across the membrane were stained with crystal violet, imaged by microscope. The crystal violet on the membrane was dissolved in 30% acetic acid, and the absorbance at 590 nm was recorded by a microplate reader.

##### Pharmacokinetics

BALB/c mice were randomly divided into 4 groups (n = 5), and intravenously injected with free Dox, HPMA‐Dox conjugate, D‐NP, and GD‐NP(TD) with an equivalent Dox dose of 2 mg kg^−1^. At predetermined time points, 50 µL whole blood was collected from the orbital and extracted by acetone to measure drug concentration in blood. Pharmacokinetic parameters can be calculated with PKSolver.

##### In Vivo Therapeutic Effects

Orthotopic metastatic breast cancer mice models were established by subcutaneous injecting 5 × 10^5^ 4T1 cells into the fat pad of the fourth breast in female BALB/c mice. When the tumor volumes reached around 100 mm^3^, the tumor bearing mice were randomly divided into different groups (*n* = 5), and intravenously given various treatments (equivalent to a Dox dose of 2.5 mg kg^−1^). Treatments started on day 0, and were performed every 3 days for 4 cycles. The body weight and tumor size were monitored every 2 days after the first treatment. At the endpoint, the tumor tissues from each group were collected and immunohistochemically stained to analyze the expression of MMP2, VEGF, and TGF‐*β*. The inhibition of tumor growth was expressed as the relative tumor weight from each group compared to the saline control. Meanwhile, metastatic nodules in lung tissues were counted to determine the inhibition degree of lung metastasis.

Another metastatic breast cancer BALB/c mouse model was established by intravenous injection with 3 × 10^5^ 4T1‐Luc cells on day 0. Three days after injection, mice were randomly divided into different groups (*n* = 5), and intravenously given various treatments (equivalent Dox dose, 2.5 mg kg^−1^). Treatments were performed every 3 days for 4 cycles. On day 21, mice were intraperitoneally injected with d‐luciferin potassium salt (15 mg mL^−1^, 200 µL), prior to imaging the in vivo bioluminescence in mice and ex vivo bioluminescence in extracted lung tissues. Hematoxylin–eosin histology analysis of lung lobe sections from mice and calculation of metastatic nodules on surface of lungs were also carried out. All work performed on animals was in accordance with and approved by the University Committee on Use and Care of Animals at the Sichuan University.

##### Statistical Analysis

Results were presented as means ± standard deviations (SD). Statistical analysis was calculated by ANOVA analysis using SPSS 22.0 software. *p*‐value < 0.05 was recognized as statistically significant.

## Conflict of Interest

The authors declare no conflict of interest.

## Supporting information

Supporting Information
