# Supplementary material for: Targeting the Opening of Mitochondrial Permeability Transition Pores Potentiates Nanoparticle Drug Delivery and Mitigates Cancer Metastasis
Source: Adv Sci (Weinh). 2020 Dec 31;8(4):2002834. doi: 10.1002/advs.202002834 (PMC7887600; doi:10.1002/advs.202002834)
Supplement: Supplementary file 1 — Supporting Information [file ADVS-8-2002834-s001.pdf]

© 2020 Wiley-VCH GmbH

ADVANCED SCIENCE
Open Access

Supporting Information

for *Adv. Sci.,* DOI: 10.1002/advs.202002834

Targeting the Opening of Mitochondrial Permeability Transition Pores Potentiates Nanoparticle Drug Delivery and Mitigates Cancer Metastasis

*Xi Lin, Lian Li, Shujie Li, Qiuyi Li, Dandan Xie, Minglu Zhou, Yuan Huang**

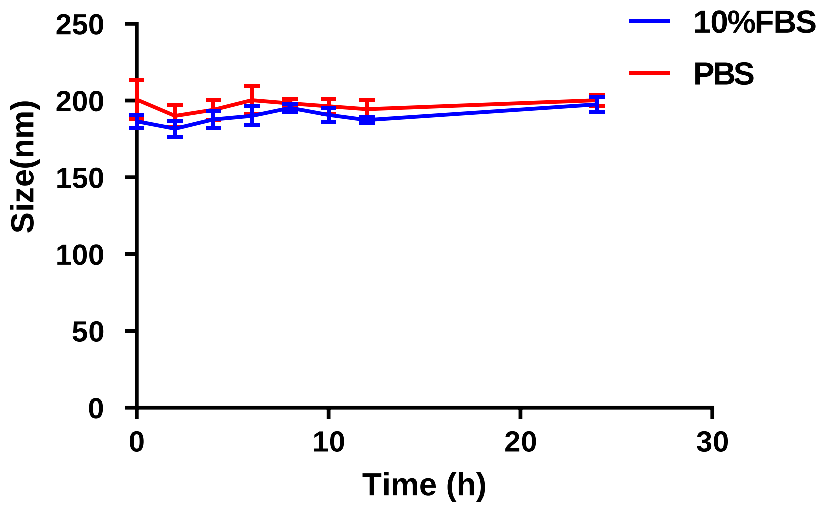

Figure S1. Particle size of GD-NP(TD) after incubation in PBS and cell culture medium containing 10% fetal bovine serum within 24 h. The stability of GD-NP(TD) was examined by dynamic light scattering (DLS). 1mL of nanoparticle at a 1 mg/ mL concentration was incubated at 37°C and 90 rpm under various conditions (PBS or 10% FBS). At predetermined time points, the size of GD-NP(TD) was determined using DLS.

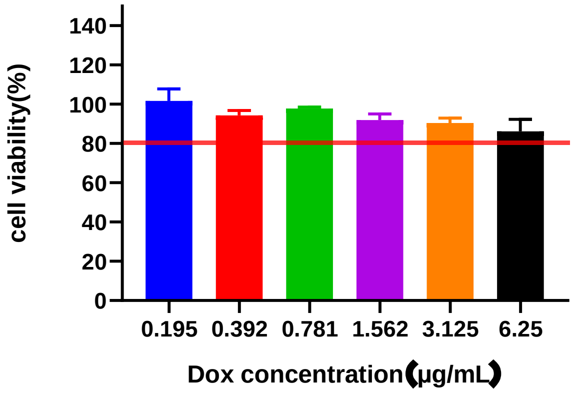

Figure S2. Cytotoxicity of GD-NP(TD) on normal human umbilical vein endothelial cells (HUVEC) with a series of Dox concentration for 24 h incubation.

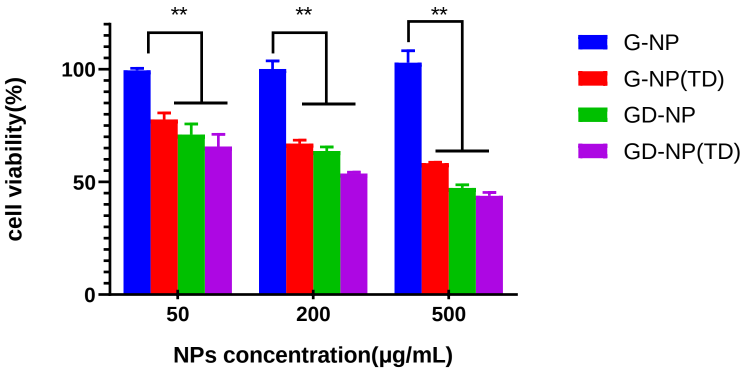

Figure S3. Viabilities of 4T1 cells treated with various nanoparticles for 24 h incubation ,n=3; *P<0.05; **P<0.01.

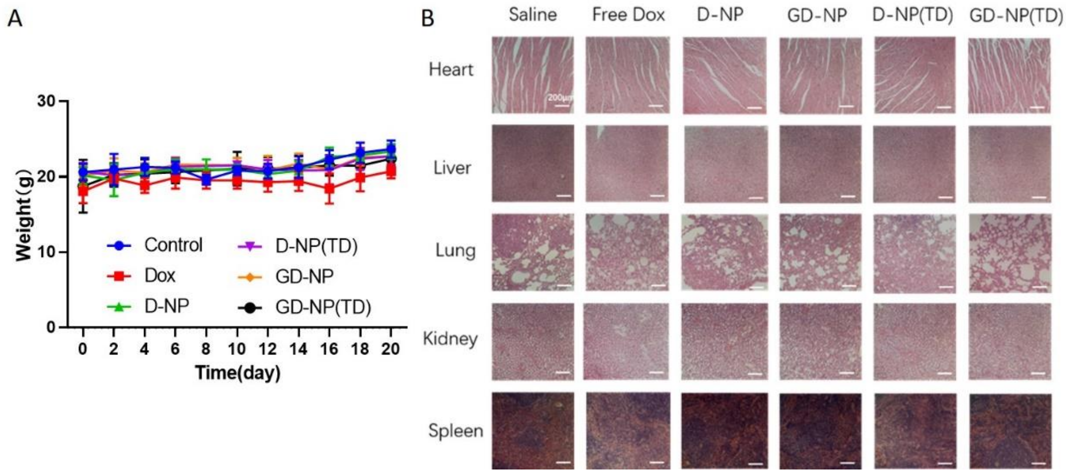

Figure S4. (A) Changes of body weight among all the groups during the treatment. (B) Hematoxylin–eosin histology analysis of major organs in 4T1 tumor-bearing mice treated with different groups at the endpoint.
